# Effects of Fermented *Radix puerariae* Residue on Nutrient Digestibility and Reproductive Performance of Sows

**DOI:** 10.3389/fnut.2021.715713

**Published:** 2021-08-30

**Authors:** Zhenfu Luo, Yuanyuan Zhao, Liming Zeng, Jie Yin, Qinghua Zeng, Xilong Li, Jianhua He, Jing Wang, Bi'e Tan

**Affiliations:** ^1^College of Animal Science and Technology, Hunan Agricultural University, Changsha, China; ^2^College of Animal Science, Jiangxi Agricultural University, Nanchang, China; ^3^Key Laboratory of Feed Biotechnology, The Ministry of Agriculture of the People's Republic of China, Beijing, China

**Keywords:** fermented *Radix puerariae* residue, nutrient digestibility, reproductive performance, sows, offspring

## Abstract

This study was conducted to investigate the effect of fermented *Radix puerariae* residue (FRPR) on reproductive performance, apparent total tract digestibility (ATTD) of nutrients, and fecal short-chain fatty acid (SCFA) contents of sows. A total of 36 landrace × large white multiparous sows were randomly arranged into three treatments, representing supplementation with 0, 2, and 4% FRPR to a corn-soybean meal and wheat bran-based diet during the whole gestation period. The results showed that dietary FRPR had no effects on litter size and the number of total alive piglets (*P* > 0.05), and that the number of weaned piglets and weaning weight of litter were increased in sows with 4% FRPR treatment compared with control treatment (*P* < 0.05). Dietary 4% FRPR significantly decreased constipation rate, improved the ATTD of dry matter and organics, and fecal contents of acetate, propionate, and total SCFAs (*P* < 0.05). In the offspring piglets, serum concentrations of total protein, alkaline phosphatase, IgG, IL-10, and TGF-β were increased, but blood urea nitrogen content was decreased with 4% FRPR treatment (*P* < 0.05). There were no significant differences in all determined indexes except for fecal acetic acid and total SCFAs between control and 2% FRPR treatment (*P* > 0.05). These findings indicated that FRPR used in the diets of sows showed positive effects on fecal characteristics, utilization of nutrients, and reproductive performance. Maternal supplementation with 4% FRPR is recommended for improving immune responses, weaning litter size, and litter weight of offspring piglets, which provide useful information for the application of residues of *R. puerariae*.

## Introduction

*Pueraria lobata Ohwi* (Latin name *Radix puerariae*), a Chinese traditional medicine, has always been used to treat diabetic nephropathy, cardiovascular diseases, Parkinson's disease, Alzheimer's disease, and acute cerebral ischemic stroke (1). Its roots contain many kinds of isoflavones, triterpenes, saponins, and so on. After the extraction of these substances, there are still lots of cellulose and some active components that remain in residues. The dregs of *R. puerariae* have been determined to contain 4.79% crude protein, 58.08% neutral detergent fiber, 22.96% acid detergent fiber, and 1.18% flavonoids. More than 18 tons/acre of *R. puerariae* are produced, but millions of tons of residues are discarded, which is a huge waste and can also lead to serious pollution. Fermentation is an important way of boosting the comprehensive utilization of the residue of *R. puerariae* and other feeds (2). Fermented *Radix puerariae* residue (FRPR) may be an unconventional feedstuff for livestock, especially as a fiber source in sows, due to its possible effects on gut health and welfare.

Under commercial intensive feeding conditions, pregnant sows are often limit-fed to prevent excessive weight gain, which can lead to negative consequences on locomotion, farrowing, and post-farrowing feed intake (3, 4). However, this kind of restricted feeding strategy may induce aggression and stereotypies (5), or even affect the health of offspring piglets. There is evidence that fiber-rich diets could increase the satiety of pregnant sows and, thereby, reduce restricted feeding-induced behavioral problems (6, 7). Moreover, fiber-rich diets for sows during pregnancy could also reduce agonistic interactions among piglets (7). Except for the benefits of animal welfare, fiber-rich diets during gestation can also improve the reproductive performance of sows and the growth performance of piglets (8, 9). Moreover, a fiber-rich diet for gestating sows during transition reduced the proportion of stillborn piglets and mortality of total born piglets (10).

Studies have shown that the nutritional composition of colostrum can be changed with additives or fiber type and content in the diet of pregnant and lactating sows, thereby regulating the growth performance and immunity of offspring piglets (11–13). In view of that, this study aims to determine the effects of dietary FRPR on reproductive performance, apparent total tract digestibility (ATTD) of nutrients, and short-chain fatty acids (SCFAs) of sows. Serum biochemical parameters and cytokine concentrations of offspring piglets are also examined to evaluate the health status of piglets after maternal dietary supplementation of FRPR.

## Materials and Methods

### Fermented *Radix puerariae* Residue Preparation

Dietary fermented *Radix puerariae* residue was prepared using residues from the roots of *Pueraria lobata* (Wild) *Ohwi* at the College of Animal Science, Jiangxi Agricultural University (Nanchang, Jiangxi, China). Residues from roots of *Radix puerariae* were autoclaved at 121°C for 1.5 h and inoculated with a liquid strain of *Trichoglossum* spp. after cooling. They were cultured at 25–27°C in a dark room for 25 days, and then dried and pulverized. The final FRPR contained 12.5% crude protein, 38.1% crude fiber, and 7.75% soluble fiber analyzed using Association of Official Agricultural Chemists (AOAC) methods.

### Animals and Experimental Design

This animal study was reviewed and approved by the Institutional Animal Care and Use Committee of Hunan Agricultural University, Hunan, China. The animal protocol was approved by Institutional Animal Care and Use Committee (IACUC No. 20190056). Thirty-six 3–4 parity Landrace × Large White multiparous sows housed in individual stalls were randomly assigned into three treatments (control, 2% FRPR, and 4% FRPR) with 12 sows/treatment and fed with diet supplementation with 0, 2, and 4% FRPR, respectively. The diets were formulated to meet the nutrient requirements for the early and late stages of pregnancy for sows and contain equal nitrogen and energy (Table 1). Wheat bran of 0, 2, and 4% were replaced by FRPR, and the final crude fiber contents were 5, 5.55, and 6.15% (days 0–84 of gestation), and 3.5, 4.1, 4.7% (days 85–114 of gestation). The sows were fed with individual diet twice a day based on their body weight as follows: 1.7–2.2 kg/days from mating day to 30th day of gestation, 2.2–2.5 kg/days from 31st to 84th day of gestation, and 2.8–3.5 kg/days from 85th day of gestation to parturition day. On day 107 of gestation, all the sows were moved into an environmentally controlled farrowing room equipped with a feeder and a nipple drinker for sows, and as a nipple drinker and a heating lamp were provided for suckling piglets. All lactating sows were fed the same lactation diet and piglets were fed the same creep feed *ad libitum* until weaning at 21 days. All the sows and piglets had free access to water during the whole experimental period.

**Table 1:** Ingredient composition of diets in early and later stages of pregnancy.

**Items**	**Day 0–84 of gestation**			**Day 85–114 of gestation**		
	**Control**	**2%FRPR**	**4%FRPR**	**Control**	**2%FRPR**	**4%FRPR**
**Ingredient, %**						
Corn	52.80	53.00	52.80	63.80	63.50	63.20
Soybean meal (43% CP)	11.60	11.40	11.60	23.50	23.70	23.90
Wheat bran	30.00	28.00	26.00	4.00	2.00	-
Puerarin fiber	/	2.00	4.00	/	2.00	4.00
Lithopone powder	1.30	1.30	1.30	1.00	1.00	1.00
Calcium hydrogen phosphate	1.20	1.20	1.20	1.20	1.20	1.20
Sodium chloride	0.40	0.40	0.40	0.40	0.40	0.40
Sodium bicarbonate	0.20	0.20	0.20	0.20	0.20	0.20
Soybean oil	0.50	0.50	0.50	1.90	2.00	2.10
Premix^*^	2.00	2.00	2.00	2.00	2.00	2.00
Fish meal	/	/	/	2.00	2.00	2.00
Total	100.00	100.00	100.00	100.00	100.00	100.00
**Nutrient** ^**^						
Digestible energy (DE, Mcal/kg)	2.93	2.93	2.93	3.34	3.35	3.34
Net energy (NE, Mcal/kg)	2.21	2.20	2.20	2.50	2.50	2.50
Crude protein (CP, %)	13.60	13.51	13.51	17.02	17.02	17.02
Crude fiber (CF, %)	5.00	5.55	6.15	3.50	4.10	4.70
Lystine (Lys, %)	0.70	0.70	0.70	1.08	1.09	1.09
Methionine (Met, %)	0.23	0.22	0.22	0.41	0.41	0.41
Tryptophan (Try, %)	0.17	0.16	0.16	0.24	0.24	0.24
Calcium (Ca, %)	0.88	0.89	0.88	0.87	0.87	0.88
Total phosphorus (TP, %)	0.66	0.66	0.66	0.58	0.58	0.57
Non-phytate phosphorus (NP, %)	0.35	0.35	0.35	0.38	0.38	0.38

* *Premix was different between pre-pregnancy and late pregnancy. The premix for pre pregnancy provides 15 mg/kg Cu, 150 mg/kg Fe, 95 mg/kg Zn, 60 mg/kg Mn, 0.45 mg/kg Se, 11,000 IU/kg VA, 2,000 IU/kg VD_3_, and 100 mg/kg VE. The premix for late pregnancy provides 15 mg/kg Cu, 125 mg/kg Fe, 90 mg/kg Zn, 60 mg/kg Mn, 0.45 mg/kg Se, 7,000 IU/kg VA, 2,000 IU/kg VD_3_, and 170 mg/kg VE*.

** *Values of nutrients are calculated*.

On day 77 of gestation, 0.3% of chromium trioxide was added to the diet as an indicator to determine nutrient digestibility. After a 3-day adaptation period, fecal samples were collected for 5 days. All fecal samples from the same sow were mixed, dried at 65°C, and pulverized for chemical composition detection, and fresh fecal samples of sows were collected by rectal massage on day 84 of gestation for the analysis of SCFA concentrations. Blood samples were collected from 21-day-old piglets from the jugular vein, and serum samples were obtained by centrifugation at 3,000 × g for 10 min at 4°C and then immediately stored at −80°C for further analysis.

### Constipation Rate and Reproductive Performance of Sows

The judgment criteria for constipation in sows is dry, hard, and ball feces, which is manifested by loss of water, reduction in volume, change in texture, and darkening of color. Constipation rate in each treatment every day during the whole pregnancy period was calculated as follows: constipation rate (%) = 100 × number of constipated sows/total number of sows. Reproductive performance, which included litter size, alive litter size, number of weaning piglets, litter weight of birth, and weaning on day 21 age, was recorded and calculated.

### Apparent Total Tract Digestibility of Nutrients

Feed and fecal samples were analyzed for dry matter, total energy, crude protein, neutral detergent fiber (NDF), acid detergent fiber (ADF), organics, and crude fat according to AOAC methods. The concentration of chromium trioxide in the diet and fecal samples was measured photometrically and used to calculate nutrient digestibility.

### Fecal SCFA Concentrations in Sows

Concentrations of fecal short-chain fatty acidss, such as acetic, propionic, butyric, isobutyric a, valeric, and isovaleric, were analyzed as described in the previous study (14).

### Serum Biochemical Parameters and Cytokines Concentrations in Piglets

Serum immunoglobulins (IgG and IgM), as well as biochemical parameters (total protein, albumin, blood urea nitrogen, glucose, alanine transaminase, aspartate aminotransferase, alkaline phosphatase, triglyceride, and cholesterol) of the piglets were determined using a biochemical analytical instrument (Beckman CX4, Beckman Coulter Inc., Brea, CA, United States) and commercial kits (Sino-German Beijing Leadman Biotech Ltd., Beijing, China). Concentrations of interleukin-1β (IL-1β), IL-6, IL-8, IL-10, IL-12, tumor necrosis factor-alpha (TNF-α), transforming growth factor-beta (TGF-β), and interferon-gamma (IFN-γ) in the serum of piglets were measured using Porcine Cytokine Array Q1 (QAP-CYT-1) (RayBiotech, Inc., Guangzhou, China) according to the instructions of the manufacturer. The average value of piglets in the same litter was analyzed as a biological duplication.

### Statistical Analysis

Data were analyzed by ANOVA using the SPSS 17.0 software (SPSS, Inc., Chicago, IL, United States). Tukey's test was performed for multiple comparisons. The results were expressed as mean ± SEM, and statistically significant differences were assumed with *P* < 0.05.

## Results

### Effect of Dietary FRPR Supplementation on Reproductive Performance of Sows

As shown in Table 2, there are no constipated sows in 4% FRPR treatment, while the constipation rates of 2% FRPR treatment and control treatment are 20 and 40%, respectively. The number of weaning piglets and litter weight at the age of 21 days were increased in 4% FRPR treatment compared with control treatment (*P* < 0.05). Dietary supplementation with FRPR did not influence litter size, alive litter size, and litter weight at birth (*P* > 0.05).

**Table 2:** Effect of dietary supplementation with fermented *Radix puerariae* residue (FRPR) on reproductive performance of the sows^1^.

**Items**	**Treatment**			**SEM**	***P*-valve**
	**Control**	**2% FRPR**	**4% FRPR**		
Average constipation rate, %	40	20	0	/	/
Litter size	11.25	11.85	11.95	0.50	0.616
Alive litter size	10.40	11.30	11.80	0.41	0.088
Number of weaning piglets on day 21 age	9.80^b^	10.85^ab^	11.35^a^	0.41	0.046
Litter weight of birth, kg	16.24	16.71	16.93	2.16	0.974
Litter weight of weaning piglets on day 21 age, kg	62.24^b^	68.45^ab^	70.95^a^	2.38	0.045

1 *Values are mean ± SEM; n = 12*.

a,b *Values with different letters within the same row are significantly different (P < 0.05)*.

### Effects of Dietary FRPR on ATTD of Nutrients in Diet of Sows

The digestibility of most nutrients, such as total energy, crude protein, neutral detergent fiber, acid detergent fiber and crude fat, was not significantly different among all the three treatments (*P* > 0.05). Dietary 4% FRPR supplementation significantly increased the digestibility of dry matter and organics (*P* < 0.05), but no significant differences were found between the 2% FRPR and control treatments (*P* > 0.05) (Table 3).

**Table 3:** Effects of dietary supplementation with FRPR on nutrients digestibility of sows^1^.

**Items**	**Treatments**			**SEM**	***P*-valve**
	**Control**	**2% FRPR**	**4% FRPR**		
Dry matter, %	78.97^b^	80.48^ab^	83.97^a^	1.37	0.047
Total energy, %	88.76	89.21	90.15	1.85	0.938
Crude protein, %	87.68	87.54	89.45	1.17	0.522
NDF, %	82.57	83.57	82.46	1.127	0.801
ADF, %	79.85	81.24	78.59	0.99	0.207
Organics, %	87.59^b^	88.69^ab^	91.89^a^	1.20	0.046
Crude fat, %	56.57	56.78	57.67	0.71	0.561

1 *Values are mean ± SEM; n = 12*.

a,b *Values with different letters within the same row are significantly different (P < 0.05)*.

### Effects of Dietary FRPR Supplementation on Fecal SCFA Concentrations of Sows

The results for fecal SCFAs (acetate, propionate, butyrate, valerate, isobutyrate, and isovalerate) concentrations are presented in Table 4. Compared with the control treatment, fecal acetic acid and total SCFA concentrations in the sows with the 2 and 4% FRPR treatments were significantly increased (*P* < 0.05). The dietary 4% FRPR supplementation also significantly increased fecal propionic acid concentration compared with control sows (*P* < 0.05).

**Table 4:** Effects of dietary supplementation with FRPR on short-chain fatty acid (SCFA) contents in feces of the sows^1^.

**Items**	**Treatments**			**SEM**	***P*-valve**
	**Control**	**2% FRPR**	**4% FRPR**		
Acetic acid, mg/g	2.82^b^	4.87^a^	5.97^a^	0.54	0.001
Propionic acid, mg/g	1.79^b^	2.67^ab^	4.64^a^	0.55	0.006
Butyric acid, mg/g	1.38	1.78	1.97	0.39	0.606
Valeric acid, mg/g	0.44	0.67	0.75	0.09	0.052
Isobutyric acid, mg/g	0.29	0.38	0.39	0.06	0.468
Isovaleric acid, mg/g	0.67	0.69	0.78	0.09	0.670
Total SCFAs, mg/g	7.39^b^	11.06^a^	14.5^a^	1.58	0.013

1 *Values are mean ± SEM; n = 12*.

a,b *Values with different letters within the same row are significantly different (P < 0.05)*.

### Effects of Maternal Dietary FRPR Supplementation on Serum Biochemical Parameters in Offspring Piglets

As shown in Table 5, compared with the control treatment, the maternal dietary 4% fermented *Radix puerariae* residue supplementation significantly increased serum concentrations of total protein, alkaline phosphatase, and IgG but decreased blood urea nitrogen in the offspring piglets (*P* < 0.05). There were no significant differences in determined serum biochemical parameters between control and the 2% FRPR treatment (*P* > 0.05).

**Table 5:** Effects of maternal dietary supplementation with FRPR on serum biochemical parameters in the offspring piglets^1^.

**Items**	**Treatments**			**SEM**	***P*-valve**
	**Control**	**2% FRPR**	**4% FRPR**		
Total protein, g/L	46.17^a^	49.34^ab^	57.14^a^	2.80	0.031
Albumin, g/L	32.14	34.24	35.48	2.65	0.676
Blood urea nitrogen, mmol/L	5.67^a^	4.87^ab^	4.08^b^	0.42	0.046
Glucose, mmol/L	6.21	6.53	6.78	0.65	0.838
Alanine transaminase, U/L	34.27	37.12	39.59	5.34	0.798
Aspartate aminotransferase, U/L	29.34	30.59	34.38	2.37	0.325
Alkaline phosphatase, U/L	201.14^b^	235.47^ab^	275.61^a^	18.62	0.037
Triglyceride, mmol/L	0.75	0.69	0.72	0.08	0.856
Cholesterol, mmol/L	2.86	2.76	2.68	0.25	0.891
IgG, g/L	1.64^b^	1.76^ab^	2.05^a^	0.11	0.038
IgM, g/L	0.46	0.51	0.49	0.09	0.940

1 *Values are mean ± SEM; n = 12*.

a,b *Values with different letters within the same row are significantly different (P < 0.05)*.

### Effects of Maternal Dietary FRPR Supplementation on Serum Concentrations of Cytokines in Offspring Piglets

**Figure 1:**
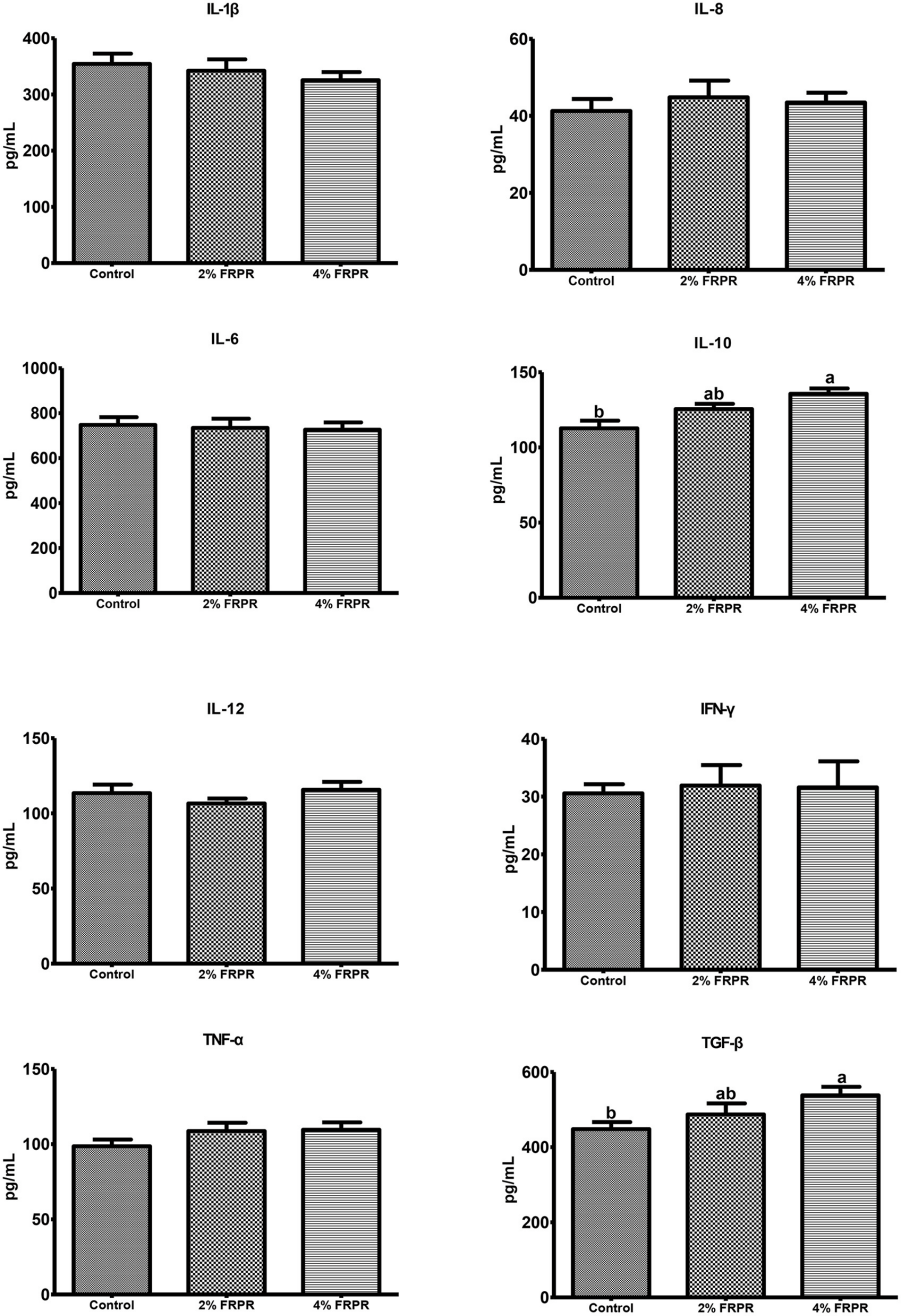
Effect of maternal dietary supplementation with FRPR on serum concentrations of cytokines in offspring piglets. Values were means ± SEM, *n* = 12. ^a,b^Values with different lowercase letters are different (*P* < 0.05).

To evaluate the immune status of the offspring piglets, serum concentrations of some cytokines were examined. The results showed that the maternal dietary 4% FRPR supplementation significantly increased (*P* < 0.05) serum IL-10 and TGF-β concentrations of the offspring piglets, but did not affect (*P* > 0.05) other inflammatory cytokines compared with the control treatment. No significant differences in cytokines were found between the piglets of 2% FRPR and control treatments (*P* > 0.05) (Figure 1).

## Discussion

The reproductive performance of sows is highly associated with profits from pig husbandry. Even a slightest increase in the number of piglets weaned per sow per year has considerable commercial advantages (8). Many studies have highlighted the beneficial effects of a high fiber diet on the reproductive performance of sows (15–20). Dietary supplementation with a by-product from *R. puerariae* is expected to improve the reproductive performance and shows beneficial effects on satiety and behavior as other fiber sources in sow (21).

Although the maternal dietary FRPR supplementation did not affect litter size and litter weight at birth, the 4% FRPR treatment significantly increased the number and litter weight of weaning piglets. These results were consistent with previous studies, such as the addition of 13.35% ground wheat straw to the gestation diet improving litter size and total litter weight at birth and weaning (5, 22). Dietary fiber has been demonstrated to improve oocyte quality and early embryo survival rate, increase litter size, prevent gestation and miscarriage, and improve the reproductive performance of sows (16–18, 20). High dietary fiber reduced estrogen during follicular development that could improve the survival rate of early embryos and reduce intrauterine growth retardation of fetal pigs, even stimulated the immune system, and reduced diarrhea rate and mortality (15).

Based on fecal characteristics, no sows in the 4% FRPR treatment had constipation, but the sows in the control treatment had a 40% constipation rate. Constipation may exaggerate the release and absorption of bacterial endotoxins and, thereby, the development of postpartum dysgalactia syndrome in sows (23). Another serious consequence of constipation is increased farrowing duration of sows due to the pain and discomfort caused by constipation (24). It has been suggested that farrowing duration was directly associated with the number of stillborn (25). Sows with longer farrowing duration are at a greater risk for having fever on the first day after parturition (26). It has been reported that a high-fiber diet increases fecal water content (27), which is a mutual confirmation with the results.

Fully understanding the effects of dietary fiber on nutrient digestibility is essential for the proper usage of fiber-rich diets in gestating sows. Dietary fibers generally have a negative impact on nutrient and energy digestibility. Previous studies have revealed that sows fed with a high-fiber diet exhibited lower digestibility of dry matter, crude protein, gross energy, non-fibrous carbohydrates, and organic matter (10, 28). However, there are also some opposite results due to different dietary fiber sources and compositions, as well as physiological stages of animals. Research reported that dietary energy digestibility improved with an increase in dietary soluble fiber (29). Agyekum et al. also found that supplementing processed straw, which is rich in fiber, during late gestation improved the apparent total tract digestibility of dry matter and energy (5). The authors speculated that the processing of straw might have improved digestibility by changing the fiber matrix, reducing fiber length, and opening up the cell wall structure. In this study, the dietary 4% FRPR supplementation significantly increased the digestibility of dry matter and organics. The relatively high soluble contents (7.75%) in the FRPR and fungal fermentation process may play important roles.

Dietary fiber is generally not hydrolyzed by endogenous enzymes but fermented by microbes in the hindgut (30). Major products of dietary fiber fermentation are SCFAs (acetic acid, propionic acid, butyric acid, valeric acid, isobutyric acid, and isovaleric acid), which carry out diverse functional roles such as activating G-coupled-receptors, inhibiting histone deacetylases, and serving as energy substrates (31). Many studies have indicated that dietary fibers effectively alter intestinal microbiota composition and diversity in pregnant sows (9, 11, 21). Although in this study we failed to determine the intestinal microbial composition of sows, the results for fecal SCFAs may partially explain the regulation of dietary fiber on microbiota. In this study, the dietary 4% FRPR supplementation significantly increased fecal concentrations of acetic acid, propionic acid, and total SCFAs, which is consistent with the previous study that demonstrated that a high-fiber diet could increase fecal SCFA content (27, 32). Furthermore, the study showed that increasing dietary konjac flour, which is highly fermented by gut microbiota to produce SCFAs, linearly increased plasma SCFA concentration 4 h post prandial (33). Gut microbiota-derived SCFAs not only fuel host cells but also serve as signaling molecules between gut microbiota and extra-intestinal organs (34, 35). Notably, the recent findings showed that the gut microbiota of pregnant mice influence the immune and brain functions of offspring, raising the possibility that maternal SCFAs play a key role in the regulation of disease susceptibility during postnatal life (36, 37).

To investigate the maternal effects of dietary FRPR, serum biochemical parameters and cytokines concentrations in 21-day-old piglets were determined. The results showed that the maternal dietary FRPR supplementation influenced the metabolism and immune function of the offspring. The increase in serum total protein and decrease in blood urea nitrogen concentration indicated, to some extent, that the maternal FRPR supplementation improved protein synthesis and suppressed protein catabolism (38, 39), which were highly correlated with an increase in weaning weight of piglets. The increase in serum IgG concentration indicated that the immune status of piglets was improved by the maternal FRPR supplementation, which was further confirmed by a higher concentration of anti-inflammatory cytokine IL-10 and immune tolerance mediator TGF-β (40). Serum alkaline phosphatase activity was also increased in piglets of the FRPR treatment, verifying partially the probability for increase in the growth performance of piglets (41, 42).

Studies have identified that the isoflavone genistein could reduce intestinal cell proliferation *in vitro* and *in vivo* in piglets without affecting intestinal enzyme activity or nutrient transport (43). The contents of isoflavone in different sources of legumes feeds are significantly different, among different raw materials, which may reduce the severity of rhinovirus (RV) infections (44). Whether the effects of FRPR on sows and offspring piglets are due to isoflavones or other specific substances need further research.

## Conclusions

In conclusion, FRPR is a suitable fiber source for gestating sows to improve reproductive performance, ATTD of nutrients, and gut SCFAs production. Maternal supplementation with 4% FRPR is recommended for improving immune responses, weaning litter size, and litter weight of offspring piglets, which provide useful information for the application of residues of *Radix Pueraria* in sows.

## Data Availability Statement

The original contributions presented in the study are included in the article/supplementary material, further inquiries can be directed to the corresponding author/s.

## Ethics Statement

The animal study was reviewed and approved by Institutional Animal Care and Use Committee of Hunan Agricultural University.

## Author Contributions

ZL: investigation, visualization, and writing of original draft. YZ: writing the review. XL: resources. LZ and QZ: conceptualization and methodology. JH, BT, and JW: supervision and editing. All authors contributed to the article and approved the submitted version.

## Conflict of Interest

The authors declare that the research was conducted in the absence of any commercial or financial relationships that could be construed as a potential conflict of interest.

## Publisher's Note

All claims expressed in this article are solely those of the authors and do not necessarily represent those of their affiliated organizations, or those of the publisher, the editors and the reviewers. Any product that may be evaluated in this article, or claim that may be made by its manufacturer, is not guaranteed or endorsed by the publisher.

## Abbreviations

FRPR: fermented Radix puerariae residue
SCFAs: short-chain fatty acids
NDF: neutral detergent fiber
ADF: acid detergent fiber
Ig: immunoglobulins
IL: interleukin
TNF-α: tumor necrosis factor alpha
TGF-β: transformed growth factor beta
IFN-γ: interferon-gamma.
